# Altruism in medical education: assessing attitudes of hospital in-patients towards face-to-face contact with medical students during the COVID-19 pandemic

**DOI:** 10.1186/s12909-023-04066-x

**Published:** 2023-03-04

**Authors:** Alison Gritzner, Thomas Scurr, Catharine Pearce, Lexy Sorrell, Georgina Dalton, Joseph Solola, David Derry

**Affiliations:** 1 Derriford Hospital, University Hospitals Plymouth, Plymouth, PL6 8DH UK; 2 Faculty of Health, University of Plymouth, Plymouth, PL4 8AA UK; 3 Faculty of Medicine and Dentistry, University of Plymouth, John Bull Building, Plymouth Science Park, Research Way, Plymouth, PL6 8BT UK

**Keywords:** Medical education, COVID-19, Patient opinion

## Abstract

**Background:**

Limited research indicated patients were largely amenable to seeing medical students pre-pandemic. However, the COVID-19 pandemic has highlighted the potential risk of nosocomial transmission and harm to patients from students. Patient opinions regarding these risks remain unexplored, which impacts elicitation of informed consent. We aim to identify these, and explore whether reflection on the risks and benefits of direct student interaction influenced patients’ attitudes. For guidance, we further explored measures to reduce perceived infection risk.

**Method:**

We designed an original questionnaire for a cross-sectional study, completed by 200 inpatients from 25 wards between 18/02 and 16/03/2022 at Derriford Hospital, Plymouth. Patients in intensive care, with active COVID-19 infection or unable to comprehend the study information were excluded. The responses of a guardian were recorded for inpatients under 16. 17 questions were included - the initial question, reporting willingness to talk with and be examined by students, was repeated following nine questions exploring risks and benefits of student interaction. A further four questions addressed reducing the perceived infection risk. Data is summarised using frequencies and percentages, and with Wilcoxon signed-rank and rank-sum tests of association.

**Results:**

85.4% (169/198) of participants gave an initial positive response to seeing medical students, and despite a third of participants changing their response 87.9% (174/197) remained willing after the survey resulting in no significant change. Furthermore, 87.2% (41/47) of those who perceived themselves at severe risk of harm from COVID-19 remained happy to see students. Participants reported reassurance knowing students were: fully vaccinated (76.0%); wearing masks (71.5%); lateral flow test negative within the last week (68.0%) and wearing gloves and gown (63.5%).

**Conclusion:**

This study demonstrated the willingness of patients to engage in medical education despite recognised risks. Patient reflection on the risks and benefits of student interaction did not significantly reduce numbers willing to see students. Even those perceiving a risk of serious harm remained happy to have direct student contact – a demonstration of altruism in medical education. This suggests informed consent should include discussion of infection control measures, risks and benefits to patients and students, and offer alternatives to direct inpatient contact.

**Supplementary Information:**

The online version contains supplementary material available at 10.1186/s12909-023-04066-x.

## Background

As COVID-19 is now considered endemic [1], medical students are returning to their community and hospital placements. The traditional combined approach of ward-based and classroom-based education ensures the proficiency of core competencies as set out by the General Medicine Council (GMC) [2, 3], However, the risk of nosocomial COVID-19 transmission from medical students to patients during clinical placement needs to be considered within this new and changing medical landscape. Consistent with other viral illnesses, there will likely be a level of nosocomial infection, some of which may be transmitted by medical students. Inpatients are often more vulnerable than the general population, with increased morbidity and mortality in ‘at risk’ groups. Furthermore, the consequence of a single infected patient ripples out to neighbouring patients and staff, causing bed or ward closures, and blocking discharges and admissions.

Globally, limited prior research has shown patients are largely amenable to being seen by medical students [4–9], although often with a focus on intimate examinations rather than exploring opinions more generally [7, 8]. Limited international research assessing perceived risks of COVID-19 transmission in medical education has only considered the knowledge and behaviour of medical students [10, 11]. Many patients have freely offered their time, but the pandemic has made it clear that they could be endangering their health in service of medical education, a fact which necessitates informed consent. When interacting with patients and gaining informed consent – the process by which the risks and benefits of an interaction are explained and considered by the patient – clear and consistent guidance is needed as to how medical students can reassure patients that they have minimised their risk of transmitting COVID-19 and appraise them of the ongoing risks of nosocomial infection. The GMC has attempted to mitigate the risk of COVID-19 transmission from doctors, students and medical staff with national guidance, including vaccination [12, 13]. Notably, medical schools and their partner trusts have largely made COVID-19 vaccinations compulsory (unless contra-indicated). Other harm-reduction measures for staff and students are made by regional or local trusts, with individual medical schools offering guidance on personal protective equipment (PPE), lateral flow testing (LFT) and isolation [14].

Upon reviewing the literature, we believe the voice of patients has not always been heard, or even sought, with respect to the risks and benefits of seeing medical students, particularly during the COVID-19 pandemic. This study aims to explore what risks are salient to patients, and how they understand the benefits for students and for themselves. The primary objective was to examine the willingness of patients being seen by medical students for educational rather than clinical purposes during the pandemic. The secondary objectives explored whether attitudes change upon reflection of potential risk and harm, and what measures can be used to minimise the perceived risk and increase patient willingness to engage face-to-face with students.

## Methods

We designed an original questionnaire for a cross-sectional study to address our research aims. Our methods section was published online along with our open-access dataset prior to publishing the complete article [15].

### Recruitment

Inclusion criteria was only limited to inpatient adults and young people over 16 years of age who were able to comprehend the study information. On paediatric wards, consent was gained from a guardian, and the guardian’s response to the questionnaire recorded. Excluded inpatients were those admitted with active COVID-19 infection, those on the intensive care unit (due to the level of care needs), those in the emergency department (as these patients were largely not admitted), and patients who were unable to comprehend the study information. Patients were therefore not approached if their attending medical team felt they had significant acute or chronic cognitive impairment.

Data collection was completed between 18/02/2022 and 16/03/2022 inclusive at University Hospitals Plymouth, UK. We used purposive sampling to approximate the proportion of patients from different wards to the relative duration of placement time allocated to each specialty in clinical medical training at Peninsula Medical School. During their 3rd and 4th years of studies, medical students at University of Plymouth Faculty of Medicine and Dentistry have 60 clinical placement weeks. 37 of these involve students talking to and/or examining in-patients. The relative number of weeks spent in medical/ surgical/ obstetric/ paediatric/ mental health specialities was calculated as a fraction of the 37-week total and used to guide the time spent recruiting on different wards, to ensure participants were recruited from a range of wards. Each recruiter attended a ward under a different specialty and consulted the immediate nursing team for eligible patients to be approached. During the sampling window, we reviewed each day which wards recruiters were to attend to ensure the distribution was maintained, and continued the sampling of all eligible and willing patients until the sampling window closed. Eligible patients and guardians were approached on these wards within the data collection period (*n* = 234) and invited to participate, of which 183 inpatients were surveyed along with 17 guardians of paediatric patients (total 200). This was done with the expectation that patients within different specialities might have different demographics and perception of risk, and aimed to control for these confounds and ensure results are generally applicable for medical student training.

### Questionnaire design

There were no appropriate or validated pre-existing questionnaires on literature search, therefore an original questionnaire was created. The questionnaire content was peer-reviewed by the Patient Experience & Engagement Lead for University Hospitals Plymouth and a patient representative. Their feedback was incorporated into the final version of the questionnaire. The seventeen questions used were designed as statements. Responses were collected via a combination of modified Likert scale and multiple-choice questions (questionnaire is shown in full in Appendix 1 in supplementary file). The questionnaire opened with ‘*I am happy to talk with a medical student and allow them to examine me’* to assess participants initial willingness to engage in direct medical education. The following two questions covered the participant’s COVID-19 history (vaccinations and prior infections), with a further nine questions covering areas which we felt might influence participant willingness to see medical students. These questions aimed to prompt reflection by participants on the risks of COVID-19 and the benefits of medical student interaction. We then repeated the opening question to explore the effect of this reflection on willingness to engage in medical education. The final four questions were intended to ascertain what measures would minimise participants perceived risk of COVID-19 and therefore make participants feel more comfortable about being seen by a medical student, with regards to guidance at time of data collection [12–14]. These included the use of outpatient settings, vaccination and interval LFTs by students, and the use of PPE. The full questionnaire has been included as a Supplementary file.

### Delivery and analysis

The survey was hosted online by Online Surveys and administered via tablet devices. Data collection was performed by three junior doctors and two medical students, referred to hereafter as recruiters. The medical students were on clinical placement at the time of questionnaire administration. Informed consent was gained verbally after the recruiter read a standardised opening explanation to the patient and/or guardian. Participants completed the questionnaire independently on a tablet device where able, but to ensure those with sensory impairments were able to complete the questionnaire, some participants were facilitated by recruiters. We recognised this study design is open to volunteer bias, but aimed to address this by minimising exclusion criteria, and maximising patient engagement through facilitation. We aimed to match our criteria to those used when determining which patients should be approached for educational contact with medical students while on ward placements for external validity.

Data analysis was performed using the statistics software, R version 4.1.3 [16]. All variables of interest are categorical and were therefore summarised using frequencies and percentages of non-missing data. The percentage of participants reporting that they were willing to see medical students is reported with 95% confidence intervals (CI) pre-and post-reflective questions and in a hypothetical outpatient clinic. A Wilcoxon signed-rank test was used to examine the change in willingness to see medical students after reflection on associated risks and benefits, along with the change in willingness between the second measurement and in a hypothetical outpatient clinic. A Wilcoxon rank-sum test was used to compare the initial willingness of patients to see medical students when provided the questionnaire by a Doctor or by a medical student. A *p*-value of < 0.05 is considered statistically significant and all are two-sided.

## Results

### Overview and demographics

In the questionnaire, age demographics were split into seven categories; there was an individual category for parents/guardians of Paediatric inpatients. Questionnaires were administered to inpatients on 25 wards and distributed to reflect the duration of the relevant placement during medical training (see Table 1).

**Table 1:** Participant demographics including age and gender, and breakdown of participants by specialty

Age Bracket	Number of Participants (%)
Guardian of Child in Hospital	17 (8.5)
16–35	21 (10.5)
36–55	34 (17.0)
56–65	31 (15.5)
66–75	43 (21.5)
76–85	31 (15.5)
Older than 85	23 (11.5)
**Gender**	
Male	84 (42)
Female	99 (49.5)
Guardians (gender of child not known)	17 (8.5)
**Speciality**	
General Medicine (including speciality wards such as haematology, oncology, respiratory and Care of the Elderly	116 (58)
Surgery (including orthopaedics and gynaecology)	52 (26)
Paediatrics (+ 1 child on surgical ward)	16 (8)
Obstetrics	12 (6.0)
Psychiatry	3 (1.5)
Unknown	1 (0.5)

Two hundred participants completed the questionnaire, while a further 34 inpatients (14.5%, 3–11 per recruiter) declined to participate despite meeting inclusion criteria. Reasons for refusal were not formally collected, however when given included fatigue, pain, malaise, childcare needs, and concerns over imminent medical reviews and investigations.

One hundred seventy-seven participants (89.8%) had received at least 1 COVID-19 vaccination. Fifty-two participants (26.0%) said they had previously been infected with COVID-19, while 48 (24.0%) had a confirmatory test. 120 participants (60.3%) had previously spoken to or been examined by a medical student.

### Willingness to talk with and be examined by a medical student

One hundred sixty-nine participants (85.4, 95% CI 79.5 to 89.8%) answered the opening question ‘*I am happy to talk with a medical student and allow them to examine me’* with a positive response. The positive response continued after reflective questionnaire with 174 participants (87.9, 95% CI 82.3 to 91.9%) happy to see students. The Wilcoxon signed-rank test showed that reflection on the risks and benefits of direct medical student contact did not elicit a statistically significant change in response (*p* = 0.189). At each instance this question was asked, there were missing responses from two unique participants.

**Fig. 1:**
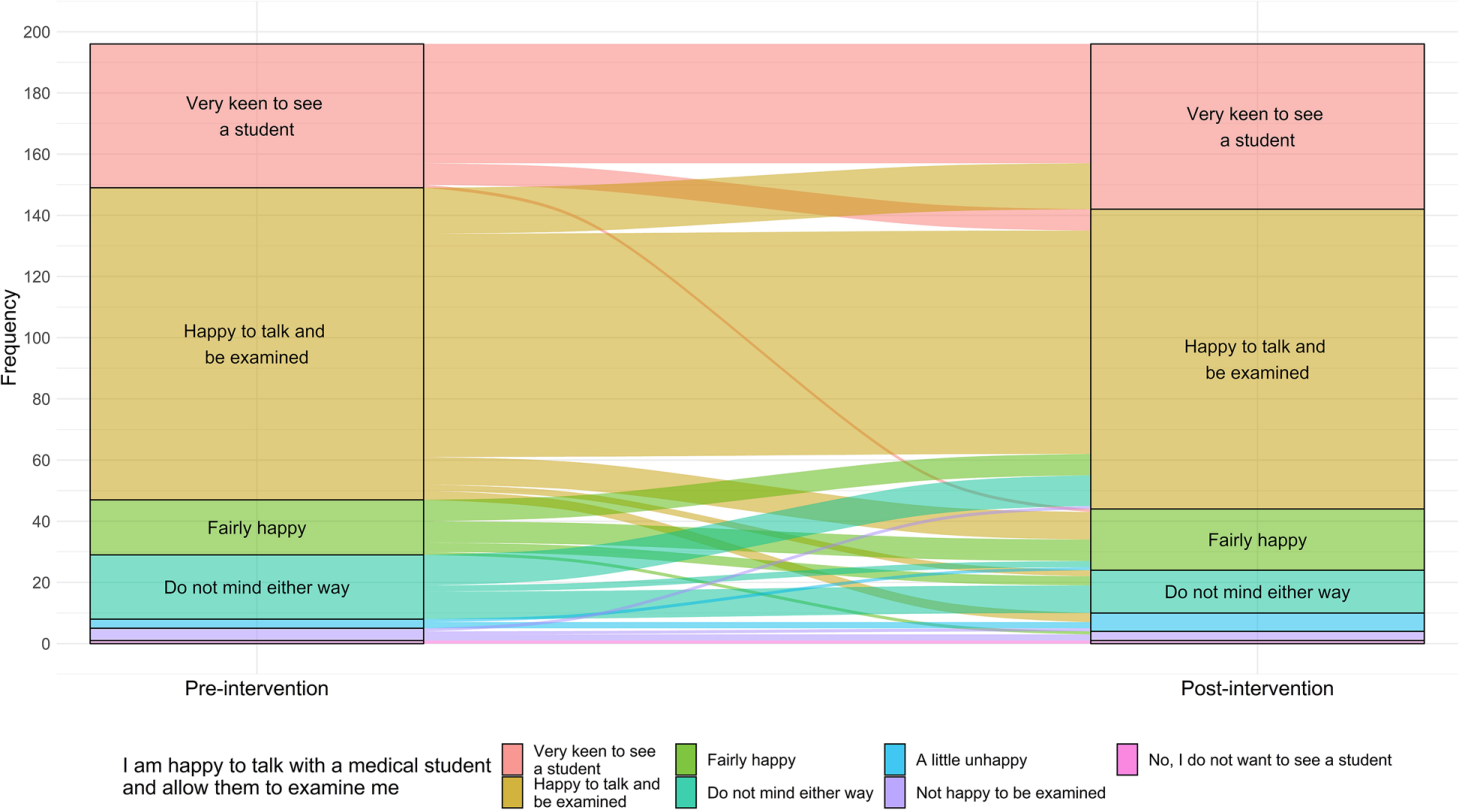
No significant change in willingness to seeing medical students after reflective questions. Mapped responses of participants response to the question *‘I am happy to talk with a medical student and allow them to examine me’* before and after the questionnaire. Analysis conducted using a Wilcoxon signed-rank test

Nonetheless, 63 participants (32.1%) did change their response (Fig. 1). Of those who changed, 37/63 participants (58.7%) changed to a more positive response and 26/63 (41.3%) to a less positive response. For the majority (40/63, 63.5%) this simply reflected a change in the degree of their original response (for example, from “fairly happy” to “happy”), rather than a change of category (e.g. from happy to neutral or unhappy). Moreover, 12/21 (57.1%) participants with an initial neutral response became happy to see students after our reflective questions, while 0/21 (0%) from the neutral group became unhappy. Of those who were initially unhappy to see students, 2/8 (25.0%) became happy. Of those who were initially happy, 5/167 (3.0%) and 4/167 (2.4%) subsequently reported being respectively neutral or unhappy to see students.

Medical students administered 77 (38.5%) of the questionnaires compared to doctors who delivered 123 (61.5%). A greater proportion of participants gave an initial positive response to seeing a medical student when the questionnaire was administered by a medical student rather than a doctor (92.1% vs. 81.1%). However, this association was not found to be statistically significant (*p* = 0.298).

### Questions to prompt consideration of the risk and benefits of seeing medical students

Questions and statements intended to explore both the clinical and educational benefits of seeing medical students elicited largely positive responses. For example, 187 participants (93.5%) agreed with the statement “*Students need to talk with patients so they can become excellent doctors in the future”,* 184 participants (92.0%) agreed that “*Students need to examine patients when they are in hospital in order to learn what people look like when they are sick*” and 168 participants (84.0%) also agreed that “*Being in hospital can sometimes be boring or scary or confusing. Talking with a medical student can be interesting and helpful*”.

Conversely, suggestions of alternatives such as “*Instead of coming to the wards, I think there are safer ways for students to practise, such as talking with patients by telephone or examining actors*” only had agreement from 32 participants (16.0%), while 144 (72.0%) disagreed with this statement. Also, respondents appeared no more concerned about *“picking up COVID”* while in hospital compared within the community (29.8% [95% CI: 23.8 to 36.7%] vs 33.6% [95% CI: 27.4 to 40.5%], respectively, responding as concerned, very concerned or extremely concerned). When asked to consider other non-clinical ward attendees, the statement “*I think the number of people coming to the ward should be tightly controlled*” showed a variety of responses with 51 (25.6%) participants agreeing with “*very limited visiting*”, whilst an equal number disagreed and selected “*anyone can visit but should COVID test beforehand*”.

After the second question self-reporting willingness, we explored how the clinical utility of student activities affected patient attitudes with the statement “*Even if junior students do not come to the wards, I think senior students should be allowed on the wards if they are helping doctors by doing tasks such as taking notes, taking blood tests and putting in cannulas”*. This demonstrated a positive response, with 185 participants (93.4%) agreeing or strongly agreeing with that statement. Four participants (2.0%) slightly or strongly disagreed that medical students should be allowed on the wards even in this capacity.

### Perception of infection risk and subsequent harm from COVID-19

**Fig. 2:**
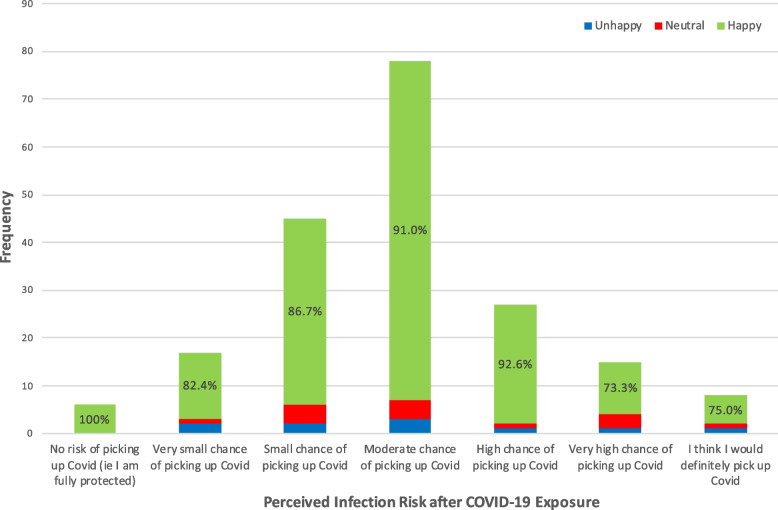
High perceived risk of contracting COVID-19 infection doesn’t significantly affect willingness to see medical students. Bar chart of participant willingness to see medical students (second question), compared to their perceived risk of contracting COVID-19 if exposed, using the statement “*If I am exposed to someone with Covid while in hospital, I think my chances of becoming infected are*..”

When asked “*If I am exposed to someone with COVID while in hospital, I think my chances of becoming infected are …*.” , participants from all self-categorised levels of perceived risk gave a highly positive response to being seen by a medical student. Of those who felt they were at the highest risk of contracting COVID-19 (responding with a ‘high chance’, ‘very high chance’ or ‘would definitely pick up COVID’), 42/50 (84.0%) were willing to see a medical student (Fig. 2).

**Fig. 3:**
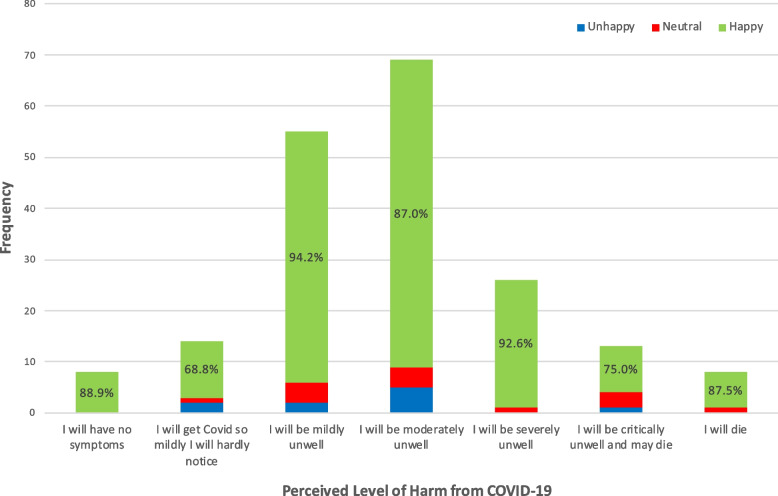
Positive responses to seeing medical students despite recognised risk of harm and death. Bar chart of participants willingness to see medical students (second question) compared to their perceived risk of harm from COVID-19 if infected, using the statement *“Because of my age and underlying medical conditions, if I do actually get Covid now, I think”*

Similarly, when asked *“Because of my age and underlying medical conditions, if I do actually get Covid now, I think …”* , over 70% of participants responded positively to seeing a medical student, across all levels of perceived harm. Moreover, in the subgroup who perceived themselves at highest risk of harm (responding ‘severely unwell’, ‘critically unwell and may die’ or ‘I will die’), 41/47 (87.2%) remained willing to be seen by a medical student (Fig. 3).

For clarity of presentation in Figs. 2 and 3, responses to the initial willingness question on the Likert scale were amalgamated into categories of; ‘happy’ (participants who selected ‘Very keen’, ‘Happy to be seen and examined’ or ‘Fairly happy’), ‘neutral’ (those who selected ‘Do not mind either way’), and unhappy (those who selected ‘a little unhappy’, ‘not happy to be examined’, or ‘no, I do not want to be seen by a medical student’).

### Reducing perceived risks - improving willingness to see medical students

Regarding alternatives to inpatient contact, 170 participants (86.7, 95% CI 81.0 to 91.0%) responded positively to being seen and examined by a medical student in outpatient clinics once their health was better. The Wilcoxon signed-rank test showed that there was not a statistically significant change in response when considering an outpatient clinic setting to the inpatient setting (second question - *p* = 0.197). However, when limited to those who were not happy about seeing medical students while an in-patient, 8/10 (80.0%) of those who were unhappy and 7/14 (50.0%) of those who were neutral about seeing students on the wards, reported being happy to see students in out-patient clinic.

**Fig. 4:**
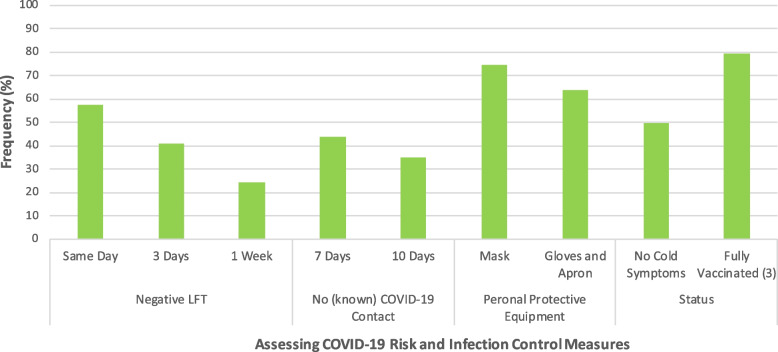
Infection control measures for medical students, as advocated for by patients. Bar chart indicating the frequency of participants who advocated for various current infection control measures for medical students (masks, gloves and apron, vaccination status and no symptoms of a cold) and the preferred measures for assessing risk of COVID-19 infection (frequency of lateral flow test and previous COVID-19 contact)

We asked participants to select other infection control measures that would make them feel more comfortable about seeing medical students, as shown in Fig. 4. They could select multiple responses regarding appropriateness of current infection control measures and the methods used for assessing risk of COVID-19 transmission, including lateral flow testing and COVID-19 contacts. Considering current infection control guidance, 152 participants (76.0%) reported they would feel happier seeing students who were fully vaccinated against COVID-19, 143 participants (71.5%) wanted medical students to wear masks, and a further 122 (63.5%) wanted students to wear gloves and gown during patient contact. Furthermore, 95 participants (47.5%) felt reassured by medical students having no symptoms of a cold. For assessing medical students’ current risk of COVID-19 infection, 110 participants (55.0%) wanted medical students to have a negative LFT result on the same day, while 136 (68.0%) wanted a negative LFT at one of the three frequencies offered. 84 (43.8%) felt reassured by knowing the medical student had not had any known contact with anyone COVID-19 positive in the last week.

## Discussion

Over 85% of participants gave a positive response when asked if they were happy to speak to and be examined by a medical student. This was irrespective of specialty, vaccination status, perceived risk of transmission from COVID-19, or perceived severity of symptoms. Participants continued to be happy to see students even when their perceived risk of infection or harm was high, demonstrating patients’ altruistic attitude towards medical education. There was also recognition that medical students need direct patient contact during their inpatient clinical attachments in order to develop the core skills needed to become excellent doctors. This overall positive response was maintained following questions exploring the risks and benefits of direct student contact to elicit informed consent. This resulted in no statistically significant difference in willingness between responses, demonstrating informed consent does not discourage engagement in medical education. A third of participants did change their response after our reflective questions and 57.1% of patients who were originally neutral in seeing medical students became positive in response. Those neutral or unhappy with direct contact were willing to see a student in outpatient clinic (50 and 80% respectively). This suggests that these questions were of value in eliciting informing consent, however conclusions are limited by the small numbers of neutral and negative responders.

Previous international published research on COVID transmission in medical education has concentrated on the risk of transmission *to* medical students [10, 11], rather than the risk *from* medical students. This has informed UK guidance for medical students, but this has not considered the anxieties this may cause inpatients [17, 18]. Prior research exploring patient attitudes towards being seen by medical students in Nigeria, Syria, Kuwait and United States of America have not hitherto been replicated in the context of the United Kingdom, nor during a pandemic [4–9]. However, our results are consistent with their pre-pandemic findings demonstrating overall positive attitudes towards direct student contact in medical education. Our study demonstrates that consideration of the risks and benefits of being seen by medical students is both ethically necessary, and beneficial to patients. Informed and engaged patients will also likely provide better educational opportunities for students.

### Limitations and future research

This study did not investigate non-COVID-19 related reasons why patients did not wish to speak to medical students, or were neutral about the fact. These reasons may overlap with the reasons given for declining to complete the questionnaire. It is likely that those who declined to participate with answering the questionnaire would also be more likely to decline to see students, so introducing a selection bias. Further exploration could evaluate participant reasons for negative attitudes to seeing medical students. However, even if *all* questionnaire non-participants were included in the number of in-patients unhappy to see students, the conclusion still remains that 169/232 (72.8%) of patients were happy to see students. Additionally, future research could benefit from investigating whether considering wider transmission of the common viral diseases, such as seasonal influenza or norovirus, influences willingness.

It may also be that having a facilitator present encouraged more positive responses, especially if these facilitators were medical students. Future studies could consider an online survey delivered remotely to remove this confounder. When designing this study, we considered whether it should be administered in this way, but felt this would bias against those with sensory or motor difficulties and those with less experience of tablet devices. Nonetheless, the consistency of our results with previous research suggests our data has external validity [4–9]. Future exploration of negative attitudes may improve our understanding of patient perception of risks.

### Recommendations

Current national guidelines recommend all medical students be fully vaccinated and wear a mask on the wards [13, 19]; this is consistent with the opinion of our participants. The second most frequently selected measure was LFT testing. Although at the time of administration of the questionnaire, regular LFT testing was a requirement for staff working in the NHS, students were not explicitly mentioned in NHS LFT provisions. Subsequently NHS-England decided that, as of 31 August 2022, LFT testing of asymptomatic staff was no longer required [20]. Our results suggest this will make participants less happy to see students; it is not known what proportion would refuse to see students without LFT testing. A majority of participants also felt reassured by students wearing gloves and gown. While their use is expected when examining patients with known infections or immunocompromise, they are not part of routine attire for students or staff. As patients become more aware of the risks of nosocomial infection, such demands for the use of additional personal protective equipment should be considered. We suggest that when seeking consent to talk with and examine inpatients, medical students should use a script which, as a minimum, clarifies:

1. the measures they have taken to minimise risk of transmission of infection that our study indicates are of importance to patients (e.g. “I have been fully vaccinated and last had a negative LFT 3 days ago.”)
2. the benefits of direct interaction for both medical students and patients (e.g. “Some medical teaching uses actors, but seeing unwell patients in hospital is important when training to become an excellent doctor, and some patients find student seeing students helpful”)
3. that there is no obligation to see students; offering an alternative (e.g. the possibility of seeing students in out-patient clinic) may make patients feel less uncomfortable about refusal, which is important for informed consent.

## Conclusion

Overall, this paper suggests the majority of patients are happy to talk with and be examined by medical students, despite a risk of COVID-19 transmission and perceived risk of serious illness or death from COVID-19. Our data demonstrates the high regard patients have for medical education, and the perceived risks and benefits for patients of engaging in medical education. The previously acknowledged goodwill of patients in engaging in clinical education has persisted, highlighting the support to future doctors during a global pandemic and despite a serious risk to health. This clearly demonstrates patient altruism in medical education.

## Supplementary Information

**Additional file 1.** Questionnaire.

## Data Availability

We are happy to share all data. Please contact the Corresponding Author for more information. The dataset generated and analysed during the current study is available in the Figshare repository from: 10.6084/m9.figshare.21029602.v1.
