# Supplementary material for: Altruism in medical education: assessing attitudes of hospital in-patients towards face-to-face contact with medical students during the COVID-19 pandemic
Source: BMC Med Educ. 2023 Mar 4;23:149. doi: 10.1186/s12909-023-04066-x (PMC9985091; doi:10.1186/s12909-023-04066-x)
Supplement: Supplementary file 1 — Additional file 1. Questionnaire. [file 12909_2023_4066_MOESM1_ESM.docx]

**APPENDIX**

**Appendix 1 – Questionnaire**

**Q1: I am happy to talk with a medical student and allow them to examine me.**

- Very keen to see a student
- Happy to talk and be examined
- Fairly happy
- Don’t mind either way
- A little unhappy
- Not happy to be examined
- No, I don’t want to see a medical student

**Q2: Have you previously been infected with COVID?**

- Yes (confirmed by test)
- Yes (I think I have had COVID, but no positive test)
- No

**Q3: Have you been vaccinated against COVID?**

- I have not been vaccinated
- Yes, I’ve had 1 x vaccine
- Yes, I’ve had 2 x vaccine
- Yes, I’ve had 3 x vaccine
- Yes, I’ve had 4 x vaccine

**Q4.1: Are you worried about picking up COVID…in the community?**

- Extremely concerned, it puts me off going out or to the hospital
- Very concerned
- Concerned
- Very slightly concerned
- Not concerned at all

**Q4.2: Are you worried about picking up COVID… while in hospital?**

- Extremely concerned, it puts me off going out or to the hospital
- Very concerned
- Concerned
- Very slightly concerned
- Not concerned at all

**Q5: Being in hospital can sometimes be boring or scary or confusing. Talking with a medical student can be interesting and helpful.**

- Strongly agree
- Slightly agree
- Neutral
- Slightly disagree
- Strongly disagree

**Q6: Students need to talk with patients so they can become excellent doctors in the future.**

- Strongly agree
- Slightly agree
- Neutral
- Slightly disagree
- Strongly disagree

**Q7: Students need to examine patients when they are in hospital in order to learn what patients look like when they are sick.**

- Strongly agree
- Slightly agree
- Neutral
- Slightly disagree
- Strongly disagree

**Q8: Instead of coming to the wards, I think that there are safer ways for students to practise, such as talking with patients by telephone or examining actors.**

- Strongly agree
- Slightly agree
- Neutral
- Slightly disagree
- Strongly disagree

**Q9: I think the number of people coming to the wards should be tightly controlled.**

- Strongly agree, only essential staff allowed on wards
- Agree, very limited visiting
- Slightly agree
- Neutral
- Slightly disagree
- Disagree. Anyone can visit, but should COVID test beforehand
- Strongly disagree. Anyone can visit

**Q10: I am concerned that lateral flow tests may not detect infectious people coming to the ward, or people may forget to do the lateral flow tests even if asked.**

- Strongly agree
- Slightly agree
- Neutral
- Slightly disagree
- Strongly disagree

**Q11: If I am exposed to someone with COVID while in hospital, I think my chances of becoming** **infected are…**

- I think I would definitely pick up COVID
- Very high chance of picking up COVID
- High chance of picking up COVID
- Moderate chance of picking up COVID
- Small chance of picking up COVID
- Very small chance of picking up COVID
- No risk of picking up COVID (i.e. I am fully protected)

**Q12: Because of my age and underlying medical conditions, if I do actually get COVID now, I think……**

- I will die
- I will be critically unwell and may die
- I will be several unwell
- I will be moderately unwell
- I will be mildly unwell
- I will get COVID so mildly I will hardly notice
- I will have no symptoms

**Q13: I am happy to talk with a medical student and allow them to examine me.**

- Very keen to see a student
- Happy to talk and be examined
- Fairly happy
- Don’t mind either way
- A little unhappy
- Not happy to be examined
- No, I don’t want to see a medical student

**Q14: I would be happy to talk or be examined by students in an outpatient clinic when my health is better.**

- Very keen to see a student
- Happy to talk and be examined
- Fairly happy
- Don’t mind either way
- A little unhappy
- Not happy to be examined
- No, I don’t want to see a medical student

**Q15: Even if junior students do not come to the wards, I think senior students should be allowed on the wards if they are helping doctors by doing tasks such as taking notes, taking blood tests and putting in cannulas.**

- Strongly agree, all students should be allowed on the ward if they are helping staff
- Slightly agree
- Neutral
- Slightly disagree
- Strongly disagree, I don’t think any students should be allowed on the wards

**Q16: Which of the following would make you feel more comfortable about seeing a medical student on the ward (tick all that apply)? If I knew the student:**

- Was fully vaccinated
- Had a negative Lateral Flow Test – that day
- Had a negative Lateral Flow Test – within 3 days
- Had a negative Lateral Flow Test – that week
- Had not been to any other hospital wards that week
- Had not been in contact with anyone with COVID in the previous week
- Had not been in contact with anyone with COVID in the past 10 days
- Had no symptoms of a cold
- Was wearing a mask
- Was wearing gloves and an apron
- Other

**Q17: Which of the following would make you feel more comfortable about seeing a medical student on the ward (indicate all that apply)?**

- If the number of people with COVID in the community was low
- If the number of people with COVID in the community was falling
- If the current strain of COVID in the community is a milder one
- If there were fewer than 3 patients with COVID in the hospital
- If there were fewer than 10 patients with COVID in the hospital
- If there were fewer than 30 patients with COVID in the hospital
- If there were fewer than 100 patients with COVID in the hospital
